# What Has the Study of the K3 and K5 Viral Ubiquitin E3 Ligases Taught Us about Ubiquitin-Mediated Receptor Regulation?

**DOI:** 10.3390/v3020118

**Published:** 2011-01-28

**Authors:** Jessica M. Boname, Paul J. Lehner

**Affiliations:** School of Clinical Medicine, Cambridge Institute for Medical Research, University of Cambridge, Cambridge, CB2 0XY, UK; E-Mail: pjl30@cam.ac.uk

**Keywords:** viral E3 ligase, E2 conjugating enzyme, ubiquitin, lysine63, lysine11, Membrane Associated RING-CH (MARCH), K3, K5, endocytosis

## Abstract

Cells communicate with each other and the outside world through surface receptors, which need to be tightly regulated to prevent both overstimulation and receptor desensitization. Understanding the processes involved in the homeostatic control of cell surface receptors is essential, but we are not alone in trying to regulate these receptors. Viruses, as the ultimate host pathogens, have co-evolved over millions of years and have both pirated and adapted host genes to enable viral pathogenesis. K3 and K5 (also known as MIR1 and MIR2) are viral ubiquitin E3 ligases from Kaposi’s Sarcoma Associated Herpesvirus (KSHV) which decrease expression of a number of cell surface receptors and have been used to interrogate cellular processes and improve our understanding of ubiquitin-mediated receptor endocytosis and degradation. In this review, we summarize what has been learned from the study of these viral genes and emphasize their role in elucidating the complexity of ubiquitin in receptor regulation.

## 1. Introduction

Our understanding of the importance of ubiquitin in the regulation of different cellular processes has increased dramatically over the past twenty-five years [1–3]. One pathway controlled by ubiquitin is the regulation of cell surface receptors and their downstream signaling partners. Indeed the complexity of the ubiquitin system allows a fine-tuning of this pathway. Ubiquitin is an evolutionarily conserved 76 amino acid protein that modifies protein substrates through the cascade activity of effector proteins. It is activated through the ATP-dependent activity of the cellular E1 protein. The activated ubiquitin is transferred to one of 37 cellular E2 or ubiquitin conjugating enzymes and then directed to its cellular target through the activity of the E3 ligase—predicted to number up to 600 per cell [4,5]. The complexity of the ubiquitin system comes from a combinatorial increase in the number of modifications. Post-translational modification by ubiquitin can occur at a single site (monoubiquitination) or at multiple sites (multiple monoubiquitination), and from the first ubiquitin modification, a chain of ubiquitin molecules can be generated (polyubiquitination) through the formation of an isopeptide bond between the C-terminal glycine of the incoming ubiquitin molecule and any one of the seven lysine residues (as well as the N-terminal methionine) of the acceptor ubiquitin. Ubiquitin conjugation through different chain linkages promotes different conformational folds, resulting in different structures [6], making the complexity of the system formidable. Ubiquitin can modify cysteines, serines and threonines as well as lysine residues. All these chains require removal or remodeling by de-ubiquitylating enzymes (DUBs), producing a system of immense complexity, itself requiring fine regulation.

Viruses are by definition obligate intracellular parasites and have co-evolved in the face of strong host immune pressure. Given the importance of ubiquitin in the host cell, it is not surprising that viruses manipulate the ubiquitin pathway to further their own replication [7]. Indeed, exploiting the ubiquitin pathway provides the virus with an excellent mechanism for disposing of unwanted elements of the immune system. A good example of this activity comes from studies of the herpesviruses, large double-stranded DNA viruses that persist for the lifetime of the host, which have evolved multiple mechanisms to evade both innate and adaptive immune responses. The identification of K3 and K5 as viral ubiquitin E3 ligases has furthered the elucidation of how Kaposi’s Sarcoma Associated Herpesvirus (KSHV) evades the host immune response, as well as providing an excellent system to study the role of ubiquitination in endocytosis.

## 2. K3 and K5 Modulate Host Cell Surface Receptors

At the turn of the century, several groups reported the downmodulation of cellular immunoreceptors by novel proteins encoded by the gamma herpesviruses [8–12]. Both K3 and K5 from KSHV downregulate cell surface MHC I molecules, as does mK3, the homologue from murine gammaherpesvirus 68 (MHV-68). Radioimmune precipitation experiments showed that expression of mK3 led to the rapid degradation of endoglycosidase H sensitive MHC I, which could be rescued by proteasome inhibition [13]. In contrast, K3 and K5 did not interfere with MHC I synthesis, assembly or egress, but caused MHC I downregulation from the cell surface by enhancing endocytosis and lysosomal degradation [9–11].

K3 and K5 show different HLA allotype specificities, as K3 downregulates HLA-A, -B, -C and HLA-E, while K5 effectively targets HLA-A and -B, HLA-C only weakly and is unable to target HLA-E [10]. While K3 appears to be focused on MHC I, K5 is much more promiscuous and is able to downregulate an increasing number of seemingly unrelated immunoreceptors, as described below.

## 3. Bioinformatic Clues to K3 and K5’s Mechanism of Action

Despite the KSHV and MHV-68 homologues K3, K5 and mK3 differing in their target specificity, as well as route of target degradation, clues as to their mechanism of action were gained from sequence analysis. All three proteins contain an N-terminal motif that shares similarities with both plant homeodomain (PHD) or leukemia-associated protein (LAP) motifs and really interesting new gene (RING) domains [14]. The order of cysteine and histidine residues in these different sequences is subtly different, resulting in a different structural organization. PHD/LAP domains are implicated in transcriptional regulation and protein-protein interactions and have a conserved C_4_HC_3_ sequence organization. RING domains have either a C_3_HC_4_ (RING-HC) or a C_3_H_2_C_3_ organization (RING-H2), and act as E3-ubiquitin ligases which direct substrates for ubiquitination. Since the RING domains of K3, K5 and mK3 do not strictly match either motif, we solved the NMR structure of the K3 RING to show that the N-terminal motif common to the gamma-herpesvirus homologues, despite having a C_4_HC_3_ motif, belonged to a new family of RING finger proteins termed RING-CH [15–17].

In addition to the N-terminal RING domain common to K3, K5 and mK3, these proteins also possess two long hydrophobic stretches predicted to encode transmembrane domains. Both N- and C-termini of mK3 are cytosolic, suggesting these new RING-CH containing proteins have a type III transmembrane topology, which is unusual for E3 ligases [13]. Therefore, bioinformatic analysis predicted that K3, K5 and mK3 are viral E3-ubiquitin ligases, but could they direct the ubiquitination of substrates?

## 4. The RING-CH Domains of mK3, K3 and K5 Direct Ubiquitination

The use of proteasome or lysosomal inhibitors in cells expressing mK3, K3 or K5 allowed the visualization of ubiquitinated MHC I species. Ubiquitination is absolutely dependent on an intact RING domain. In cells treated with lactacystin, a characteristic ‘ubiquitin ladder’ was observed when MHC I was immunoprecipitated from cells expressing wild-type mK3 but not mK3 RING mutants. Re-immune precipitation with an anti-ubiquitin antibody identified this ladder of bands as ubiquitinated MHC I [13]. Similarly, K3 ubiquitinates MHC I and mutation of a critical tryptophan in the K3 RING, which does not disrupt RING structure but prevents E2 binding, abolished MHC I ubiquitination [18]. In contrast, only K5 was able to ubiquitinate B7-2 [19].

## 5. mK3 is an ERAD E3 Ligase

The endoplasmic reticulum (ER) associated degradation or ERAD pathway allows cells to dispose of proteins that fail their quality control checkpoints and are unable to progress through the secretory pathway. Misfolded proteins are selected by chaperones and retrotranslocated back across the ER membrane to the cytosol where they are targeted for proteasome-mediated degradation. This retrotranslocation step requires the polyubiquitination of the misfolded substrate—the critical step involving mK3. By acting as a virally-encoded E3 ligase responsible for the polyubiquitination of MHC I, mK3 directs the class I to the cellular ERAD pathway with a requirement for the cellular proteins Derlin1 and p97 [20].

MK3 associates in the ER membrane with the TAP/tapasin/ MHC I peptide loading complex (PLC) where it is ideally located to ‘capture’ newly synthesized MHC I heavy chain [21]. In addition to direct ubiquitination of classical MHC I molecules, mK3 also targets TAP and tapasin resulting in impaired maturation of both classical MHC I and non-classical class I [22,23]. Indeed the stabilization of mK3 by binding the PLC affords mK3 resistance to the effects of interferon-gamma (IFNγ). As IFNγ levels increase, levels of not only the PLC but also mK3 itself increase, allowing the viral protein to target the newly synthesized MHC I for ubiquitination and degradation. While the majority of K5’s substrates are targeted for endocytosis from the plasma membrane with subsequent lysosomal degradation, K5 has also been shown to target one of its substrates, PECAM/CD31, for degradation by the proteasome [24].

## 6. Endocytosis Induced by K3 and K5

The constitutive ubiquitination directed by K3 and K5 to their target immunoreceptors provides a good model system for studying the cellular components necessary for endocytosis and degradation. Whereas treatment with proteasome inhibitors prevents the degradation of ERAD substrates, lysosomal inhibitors such as concanamycin A or bafilomycin A prevent the degradation of K3 and K5 substrates targeted via the endolysosomal pathway. While K3 predominantly targets MHC I (as above) it is also reported to downregulate CD1d [25], PECAM [26] and IFN-γR1 [27]. In contrast, K5 targets many immunoreceptors for downregulation. These include ligands for cytotoxic T cells (MHC I, as above) and the related HFE protein [28], adhesion molecules (ICAM-1, PECAM, ALCAM, and VE-cadherin) [29], costimulatory molecules (B7-2) [10], ligands for NKT cells (CD1d) [25], ligands for NK cells (MICA, MICB, AICL) [30], cytokine receptors (IFN-γR1) [27], cellular restriction factors (BST-2 or tetherin) [31–33], the plasma membrane t-SNARE syntaxin-4 [31] and a member of the TGF-beta family (BMPRII) [34].

Mutational analysis of lysines in the cytosolic tail of HLA-A2 found that K3 preferentially targets the conserved membrane distal Lys340 [18], while K5 preferentially targets the conserved membrane proximal Lys335 [35,36]. Further analyses seem to confirm the general rule that K5 ubiquitinates lysines at or near the stop transfer sequence in the cytosolic tail of its substrates [28,30,34]. This positively charged region found in the majority of type I membrane proteins prevents their ‘pullback’ into the hydrophobic environment of the phospholipid membrane. This may go some way to explaining the increased substrate range of K5 *versus* K3, but cannot be the sole factor determining K5 substrate recognition since many proteins with membrane proximal lysine residues are not targeted by K5. It is surprising that K5 can target this membrane-proximal lysine which in some cases is so close to the membrane that the positively charged residue might be predicted to be embedded within the phospholipid bilayer so as not to be available for ubiquitination. One idea is that the proximity of K5, itself a membrane protein, to its substrates may help expose the acceptor lysine for targeting. In their detailed analysis of ubiquitin acceptor sites, Cadwell and Coscoy [36] found that K5 not only prefers membrane proximal targets, as opposed to the more distal targets favored by K3, but that the juxtamembrane cytoplasmic portions of K3 and K5 themselves contribute to this target preference.

## 7. K3 and K5 Expose New Amino Acid Targets of Ubiquitination

The discovery that K3 ubiquitinates MHC I lacking any cytosolic lysines led to a search for other ubiquitin acceptors [37]. The ubiquitination reaction was sensitive to reducing agents indicating the conjugation was cysteine-mediated via a thioester bond [37]. Ubiquitination of an additional HLA-B7 mutant that lacked cytosolic lysine and cysteine residues suggested that K3 could also ubiquitinate other amino acid residues. K5 also ubiquitinates a membrane-proximal cysteine residue on MHC I [36], and mK3 can ubiquitinate substrates lacking cytosolic lysines through an oxyester linkage to the hydroxyl group on serines or threonines [38]. These findings spearheaded subsequent reports that non-lysine ubiquitination can also occur in non-viral cellular systems as reported for neurogenin degradation in *Xenopus* [39] and Hrd1-dependent ERAD of the T-cell antigen receptor α-chain [40]. The frequency of these non-lysine ubiquitin acceptor sites remains to be determined.

## 8. K3 and K5 Recruit Consecutive E2s to Polyubiquitinate MHC I Molecules via Lys63 Linkages for Endolysosomal Degradation

While it was clear how polyubiquitination by mK3 would provide a signal for MHC I dislocation across the ER membrane for proteasome-mediated degradation, more puzzling was how ubiquitin could mediate receptor downregulation from the cell surface. The explanation came from a yeast two-hybrid experiment to identify the E2 which binds K3’s RING [15]. This identified UBE2N (Ubc13), a unique E2 conjugating enzyme which conjugates Lys63-linked, as opposed to the more canonical Lys48-linked, polyubiquitin chains. Rosine Haguenauer-Tsapis’ group had previously shown in yeast that Lys63 linked polyubiquitin conjugates were required for internalization of yeast plasma membrane proteins [41] but a Lys63 linkage had not been reported for receptor internalization in higher eukaryotes. Indeed, in yeast as well as mammalian systems, some groups find that monoubiquitin provides a sufficient signal for receptor internalization [42,43]—an area which remains debated [44,45].

Using a panel of ubiquitin mutants, we showed that Lys63 linked polyubiquitin chains are required for internalization, while monoubiquitination does not provide a sufficient signal [46]. Overexpression of an ubiquitin mutant lacking Lys63 (K63R) dramatically rescued MHC I surface expression in a K3-expressing cell, and biochemical analysis showed the ladder of polyubiquitinated class I species collapsed to a monoubiquitinated form. Further analyses with a lysine-less MHC I molecule fused to lysine-less ubiquitin revealed that internalization of this monoubiquitinated substrate was poor. Therefore Lys63 linked polyubiquitinated MHC I provides the signal for internalization via the endocytic pathway. A role for Lys63 is now seen with an increasing number of cell surface receptors [47,48].

The affinity of interactions between ubiquitin and ubiquitin-binding (UBD) domain-containing receptors along the endocytic pathway is low [49]. Therefore, multiple ubiquitins may increase the avidity and stabilize the interaction between ubiquitin and its corresponding UBD [42]. It would seem likely that this could occur through either multiple monoubiquitination or, for those receptors with a limited number of available cytoplasmic lysine residues, Lys63 polyubiquitin conjugates would offer an alternative option. Structural analysis shows Lys48 ubiquitin chains adopt a closed conformation, while Lys63 ubiquitin chains are in an extended conformation [50]. This extended topology is similar to adjacent linear ubiquitin chains which, unlike the Lys48 linkage, have their hydrophobic surfaces available for binding [51]. Thus, the open conformation of Lys63 polyubiquitin conjugates, as with the multiple monoubiquitins, may increase their avidity for UBDs.

Another significant finding to emerge from the analysis of K3-mediated ubiquitin chain linkages was the identification and understanding of the cellular E2 enzymes recruited by K3. To our surprise, rather than resulting in a complete loss of polyubiquitinated MHC I, depletion of UBE2N (Ubc13) from K3-expressing cells induced a collapse of the polyubiquitinated MHC I to a monoubiquitinated MHC I species which was not efficiently internalized and remained at the cell surface [46]. In addition to reinforcing the finding that monoubiquitin provides an inefficient signal for MHC I internalization, this result implied that UBE2N (Ubc13) is a processive enzyme required for elongation of the monoubiquitinated MHC I species but was itself unable to initiate MHC I ubiquitination. Thus, the priming of MHC I with a single ubiquitin must be performed by an additional E2, which was subsequently identified as UBE2N (UbcH5). This requirement for two E2 enzymes in a polyubiquitination reaction, where the first E2 primes the substrate, and the second E2 elongates the ubiquitin chain, is now well recognized in a number of ubiquitin systems. There is also a growing body of evidence that E3’s function in partnership with a limited number of E2s *in vivo* [4], and that this partnership may dictate the resulting polyubiquitin linkage [52].

A stable substrate/E3/E2/ub complex composed of MHC I heavy chain, K3, UBE2N (Ubc13) and ubiquitin was visualized by overexpression of an Ile44 mutant (I44A) of ubiquitin [53]. In this instance, surface MHC I levels in K3 expressing cells are rescued because the K3 viral ligase is unable to discharge the ubiquitin mutant onto the MHC I substrate, resulting in a stabilization of the complex. In the presence of reducing agents, the thioester bond linking the ubiquitin mutant to UBE2N (Ubc13) is broken with resulting collapse of the complex. The use of similar ubiquitin mutants may enable the capture of transient substrate/E2/E3 complexes in the future.

## 9. Internalization of MHC I by K5 Requires Mixed Lys11 and Lys63 Linked Polyubiquitin Chains on a Single Lysine Acceptor Residue

In comparison with K3, the K5-mediated downregulation of MHC I is far less efficient though the reasons behind this still remain unclear. We analyzed the ubiquitin chain linkage generated by K5 to try and understand how K5 differs from K3 and whether this has an effect on the signals required for endocytosis. As we had previously seen with K3, UBE2D (UbcH5) was required to prime MHC I with a monoubiquitin, followed by recruitment of UBE2N (Ubc13), which elongates the ubiquitin chain. Mass spectrometric analysis of the K5-generated polyubiquitinated MHC I species showed the presence of mixed polyubiquitin chains, involving Lys11- as well as Lys63-linkages, a finding which was confirmed in a series of functional experiments using ubiquitin mutants [35]. The critical point is that since these ubiquitination events occur on a single acceptor lysine residue in the tail of MHC I, this implies the presence of mixed Lys11- and Lys63-linked polyubiquitin chains. Mixed chains have been previously described *in vitro* [54], but our results are the first *in vivo* demonstration of mixed linkage polyubiquitin chains, a finding which was recently confirmed by Ishido’s group [55]. The order of chain linkages is not possible to predict, but the use of additional ubiquitin mutants suggest a forked chain topology was more likely than simple mixed chains [35]. We do not fully understand why mixed chains are required for endocytosis. The proximity of the acceptor lysine residue to the membrane phospholipid bilayer may impose constraints on the type of ubiquitin chain which can be formed. Alternatively, novel ubiquitin-binding domains may have specificity for either Lys11 or even mixed polyubiquitin chains. Although the requirements for these mixed chains are not understood, the demonstration of mixed ubiquitin chains reveals a further layer of complexity in the ubiquitin system and challenges the widely-held assumption that polyubiquitin chains are by necessity homogenous—assumptions based mainly on *in vitro* data.

## 10. Do Other Polyubiquitin Chains Substitute for Lys63 in MHC I Downregulation?

**Figure 1.**
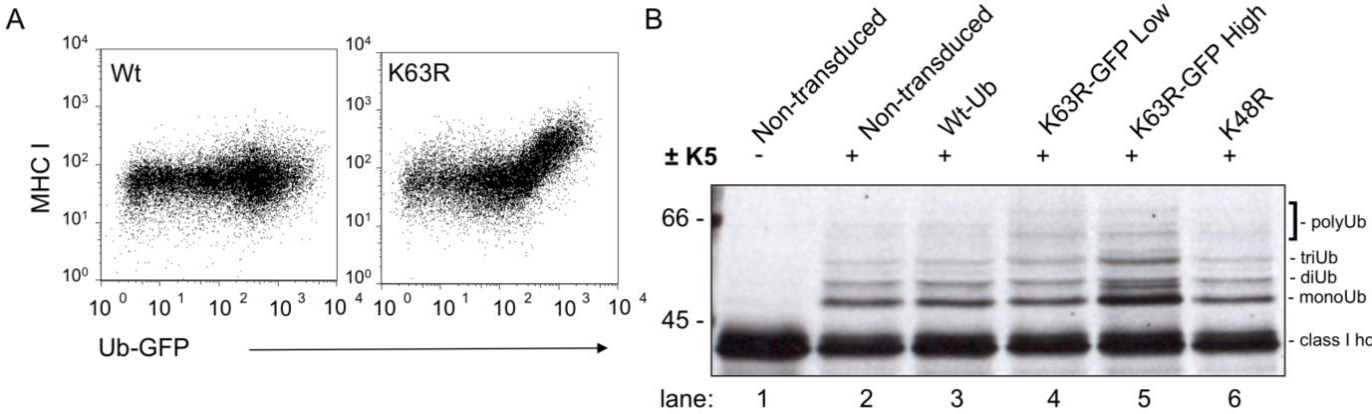
Polyubiquinated MHC I lacking Lys63 linkages is poorly downregulated. (**A**) Flow cytometry dot plot showing HeLa K5 cells transduced with ubiquitin-GFP constructs as marked. (**B**) Radioimmune precipitation showing ubiquitinated class I heavy chains. Cells were pulse labeled for 10 minutes with ^35^S-cysteine/methionine and chased for 60 minutes with unlabeled amino acids. Cells were then lysed in 1% Triton X-100 in tris- buffered saline in the presence of protease inhibitors. Cleared lysates were subjected to immunoprecipitation with w6/32, which recognizes conformational MHC I. Following denaturation at 70 °C in 1% SDS, class I heavy chain was reprecipitated with HC10 and electrophoresed on a 9% polyacrylamide gel prior to drying and exposure to film.

The role of Lys63 linked polyubiquitin chains in MHC I downregulation by K5 was established using a mutant ubiquitin, in which Lys63 was mutated to arginine (K63R) and rescued MHC I back to the cell surface as determined by flow cytometry [35]. Further biochemical analysis of this ‘rescued’ MHC I high population showed that instead of the predicted monoubiquitinated MHC I band, the MHC I in these cells showed an increase in polyubiquitinated MHC I (Figure 1B, lane 5). This result suggests (i) K5 can induce non-Lys63 linked polyubiquitin chains, and (ii) these polyubiquitin chains do not provide a signal for endocytosis. Therefore, polyubiquitination is not enough—the chain has to be the correct linkage—presumably for recognition by the correct UBD-containing protein(s).

## 11. Cellular Co-Factors Required for Endocytosis and Degradation of Polyubiquitinated Substrates

The K3-induced, Lys63-linked polyubiquitination of MHC I leads to rapid internalization and trafficking via multivesicular bodies (MVBs) for lysosomal degradation, and provides an ideal model system to identify downstream effectors required for internalization and degradation. Given that substrates decorated with Lys63-linked polyubiquitin chains by K3 are efficiently endocytosed, an siRNA approach was used to identify additional proteins required for internalization. We found an absolute requirement for clathrin and the clathrin adaptor epsin 1 [46]. Components of the endosomal sorting complex required for transport or ESCRT pathway are also required. Specifically, depletion of the ESCRT I component Tsg101 leads to the recycling of MHC I back to the cell surface in K3 positive cells because they can no longer be directed to MVBs [18], while depletion of ESCRT II components had no effect [56]. In addition, we found that the K3-mediated internalization of Lys63-polyubiquitinated class I molecules requires the epsin 1 and eps15R endocytic adaptors in a clathrin-dependent, but AP-2-independent pathway [46].

## 12. The K3 and K5 Viral E3 Ligases were Appropriated from the MARCH Family of Human E3 Ligases

RING-CH containing K3 homologues have been identified in poxviruses, where they were also found to downregulate cell surface receptors via ubiquitination [57], raising the question as to where this family of genes originated. Perhaps unsurprisingly they have been pirated from their vertebrate host. Indeed, identification of the Membrane Associated RING-CH family of E3 ligase (MARCH) proteins came from bioinformatic analyses based on the RING-CH and structural organization of K3, K5 and mK3 [16,17]. Eleven MARCH proteins can be identified in the human genome, exhibiting E3 ligase functions in different tissues with diverse substrates. The identification of substrates for the MARCH proteins remains in its infancy. Indeed, only for MARCH 1 is a physiological substrate clearly defined, as MARCH 1 regulates MHC class II expression in B cells and immature dendritic cells (DCs) [58–60]. Indeed, MARCH 1-mediated ubiquitination of MHC II in immature DCs results in degradation. Under these circumstances, ubiquitination enhances the kinetics of degradation of internalized MHC II-peptide complexes without affecting their rate of endocytosis from the plasma membrane [60]. For the remaining MARCH proteins, identification of substrates has proven more difficult as, unlike the viral ligases, protein expression is hard to detect, and no good antibodies are available. Several substrates have been identified based on overexpression of the respective MARCH protein and a search for targets identified either by (i) flow cytometry—by analogy to the known substrates of the K3 family [16,61], or (ii) a functional proteomic approach to examine the relative abundance of proteins expressed at the plasma membrane in the presence or absence of MARCH 9 [62]. Table 1 shows a summary of what is known about these cellular E3 ligases in terms of their substrates, co-factors and ubiquitin linkage specificities. These include the early identification of MHC I as a potential target of both MARCH 4 and MARCH 9 [16], to their more recent target, the NKG2D ligand Mult1 [63]. ICAM-I, FCγRIIB, SLAM, HLA-DQ, PTPRJ and ILT-2 are also potential targets of MARCH 9 identified by proteomics and confirmed by flow cytometry [62]. The proteomics approach provides a useful example of how to identify ligase substrates, but is based on the assumption that the ligase degrades its substrate—which may not always be the case.

## 13. Conclusions

In summary, the K3 family of viral ubiquitin E3 ligases enable viral survival and replication through the downregulation of a large number of cell surface receptors including MHC I, a range of immunoreceptors as well as signaling receptors and cellular restriction factors. Equally importantly, the K3 family has provided powerful tools to dissect the role of ubiquitin in receptor regulation and endocytosis, and will continue to shed light on this important pathway, for instance in the identification of cellular deubiquitinases (DUBs) and adaptors. Characterization of the K3 viral family provided the impetus for identification of the eleven human MARCH family members. Current research is focused on the identification of substrates for these cellular E3 ligases and the physiological function of MARCH family members. Many important questions about MARCH function remain, including the identity of their E2 partners, the ubiquitin chain linkage, chain length and amino acid acceptor(s). In addition, the physiological consequence of ubiquitination, including the recruitment of adaptors and fate of the substrate require further characterization.

## Figures and Tables

**Table 1.** Summary of substrates, ubiquitin acceptors, E2s, ubiquitin linkages and de-ubiquitylating enzymes (DUBs) for herpesvirus E3 ligases and their cellular homologues.

**E3 ligases**	**substrates**	**1° Ub acceptor**	**E2s**	**linkage**	**DUBs**
K3	MHC I (HLA-A, HLA-B, HLA-C, HLA-E), CD1d, PECAM, IFN-γR1	Lys or Cys 10-15 amino acids from the membrane	UBE2D (UbcH5) and UBE2N (Ubc13)	Lys63	
K5	MHC I (HLA-A, HLA-B, weakly HLA-C), HFE, CD1d, MIC-A, MIC-B, AICL, ICAM-1, PECAM, ALCAM, VE-cadherin, B7-2, IFN-γR1, Syntaxin-4, BMPRII, BST-2/tetherin	membrane proximal Lys or Cys	UBE2D (UbcH5) and UBE2N (Ubc13)	Lys63, Lys11	
mK3	MHC I, tapasin, TAP	Lys, Ser or Thr	Ube2J2 [64]	Lys48	
MARCH 1	MHC II	Lys			
MARCH 2	Transferrin receptor, B7-2, DLG1, binds syntaxin 6 [65]				
MARCH 3	binds syntaxin 6 [66]				
MARCH 4	MHC I, CD4, Mult1 (mice) [63]	Lys		Lys63	
MARCH 5	Drp1 [67]				
MARCH 6 (TEB4)	ERAD ligase [68]				
MARCH 7	unknown				USP7, USP9X [69]
MARCH 8	MHC II, B7-2, Transferrin receptor	Lys			
MARCH 9	MHC I, ICAM-1, CD4, FCγRIIB, CD150, HLA-DQ, PTPRJ, ILT-2, Mult1 (mice)	Lys			
MARCH 10	unknown				
MARCH 11	binds Veli-3 [70]				
